# *YAP1::TFE3*–rearranged inflammatory spindle cell PEComa of the anterior mediastinum in a 5-year-old child: first pediatric case

**DOI:** 10.1007/s00428-026-04470-w

**Published:** 2026-03-06

**Authors:** Rola H. Ali, Doaa Hamdy, Ahmed Nasr, Nisreen Khalifa, Abbas Agaimy

**Affiliations:** 1 Department of Pathology, College of Medicine, Kuwait University, Jabriya, Kuwait; 2 Histopathology Laboratory, Sabah Hospital, Sabah Medical District, Shuwaikh, Kuwait; 3 Department of Radiology, Chest Disease Hospital, Sabah Medical District, Shuwaikh, Kuwait; 4 Department of Pediatric Oncology, NBK Children’s Hospital, Sabah Medical District, Shuwaikh, Kuwait; 5 Institute of Pathology, Erlangen University Hospital, Friedrich Alexander University of Erlangen-Nuremberg (FAU), Krankenhausstrasse 8-10, 91054 Erlangen, Germany; 6 Comprehensive Cancer Center, European Metropolitan Area Erlangen-Nuremberg (CCC ER-EMN), Erlangen, Germany

**Keywords:** PEComa, *TFE3* rearrangement, *YAP1-TFE3* fusion, Clear cell stromal tumor of the lung, Epithelioid hemangioendothelioma, Hemangioblastoma-like tumor

## Abstract

Perivascular epithelioid cell tumors (PEComas) are rare mesenchymal neoplasms characterized by myomelanocytic differentiation and dichotomic molecular pathogenesis (mTOR-activating mutations versus *TFE3* gene rearrangements). A recently recognized subset harboring *YAP1::TFE3* gene fusion, termed inflammatory spindle cell PEComa, has been described predominantly in the lungs of adults showing significant morphologic and molecular overlap with *YAP1::TFE3*–fused clear cell stromal tumor of the lung (CCST-L). We report the first pediatric case of *YAP1::TFE3*–rearranged inflammatory spindle cell PEComa arising in the anterior mediastinum of a 5-year-old child. The tumor, largely enclosed by a thick fibrous capsule, was composed of plump ovoid to spindle cells with a prominent chronic mononuclear inflammatory infiltrate and strikingly extensive multifocal stromal dystrophic calcifications that were radiologically conspicuous. Worrisome features included large size (8 cm) and multifocal necrosis. Tumor cells showed diffuse expression of melanocytic markers (HMB45 and melanoma cocktail), cathepsin K and TFE3 in the absence of smooth muscle markers. The C-terminal YAP1 staining revealed complete loss limited to the neoplastic cells. This case expands the clinicopathologic and age spectrum of *YAP1::TFE3*–rearranged PEComa which needs to be distinguished from other overlapping neoplasms carrying the same gene fusion.

## Introduction

Perivascular epithelioid cell neoplasms (PEComas) are a rare family of mesenchymal tumors characterized by distinctive myomelanocytic differentiation. Most conventional PEComas are driven by activation of the mTOR pathway, typically through loss-of-function alterations in tuberous sclerosis complex subunits *TSC1* or *TSC2* [1]. A smaller, molecularly distinct subset harbors *TFE3* gene rearrangements, which are mutually exclusive with *TSC1/2* inactivation and involve a variety of fusion partners [2–4]. Until recently, the *YAP1::TFE3* fusion has been limited to a rare subset of epithelioid hemangioendothelioma. The specificity of this fusion was recently challenged by its identification in the majority of clear cell stromal tumors of the lung (CCST-L; initially reported as hemangioblastoma-like tumor).

In 2025, Kojima et al. reported the first two pulmonary PEComas harboring a *YAP1::TFE3* fusion, characterized by spindle cell morphology and substantial intratumoral chronic inflammatory infiltrates, and proposed the designation “inflammatory spindle cell PEComa” [5]. These tumors show significant morphologic and molecular overlap with CCST-L, an entity also defined by recurrent *YAP1::TFE3* fusion but lacking myomelanocytic differentiation [6–9]. To date, *YAP1::TFE3*–rearranged PEComa has not been reported in early childhood. Herein, we describe an intrathoracic inflammatory spindle cell PEComa with *YAP1::TFE3* fusion in a 5-year-old girl, expanding the age spectrum of this emerging PEComa variant.

## Case presentation

**Fig. 1:**
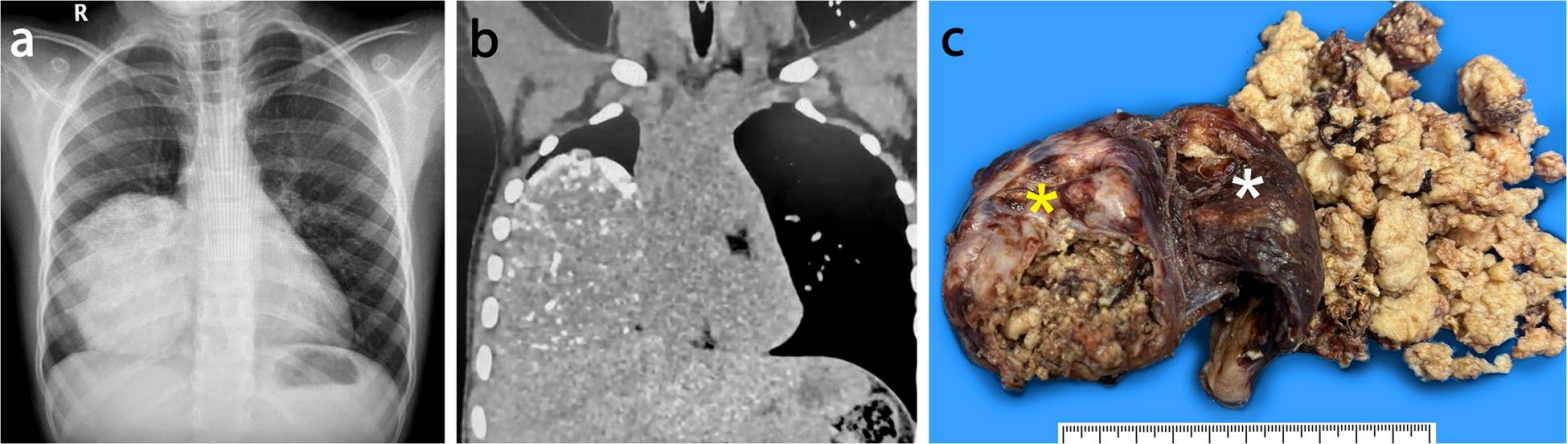
Radiologic and gross findings. **a**, **b** Chest radiograph and computed tomography (CT) showing a large, heterogeneous anterior mediastinal mass with extensive calcifications. **c** Gross specimen demonstrating the tumor (yellow asterisk) adherent to lung tissue (white asterisk), with surgically induced detached fragments

A previously healthy 5-year-old girl, evaluated for persistent cough and weight loss, underwent chest radiography, which demonstrated a large, well-defined rounded opacity occupying most of the lower half of the right hemithorax, with loss of the right cardiac border (silhouette sign) (Fig. 1a). Computed tomography revealed a heterogeneous, extensively calcified anterior mediastinal mass exerting significant mass effect, with collapse of the medial segment of the right lung middle lobe and the posterior basal segment of the lower lobe (Fig. 1b). Magnetic resonance imaging further characterized the lesion as a well-circumscribed, encapsulated anterior mediastinal mass measuring approximately 8 × 7 × 8 cm, with heterogeneous enhancement and marked vascularity. No intrapulmonary nodules or lymphadenopathy were identified. The radiologic differential diagnosis included teratoma/germ cell tumor, pleuropulmonary blastoma, and Ewing sarcoma.

Surgical excision of the anterior mediastinal mass was performed. Gross examination showed a friable tumor measuring 8 cm in greatest dimension, adherent to a wedge of lung tissue, accompanied by surgically induced detached tumor fragments (Fig. 1c).

## Morphologic findings

**Fig. 2:**
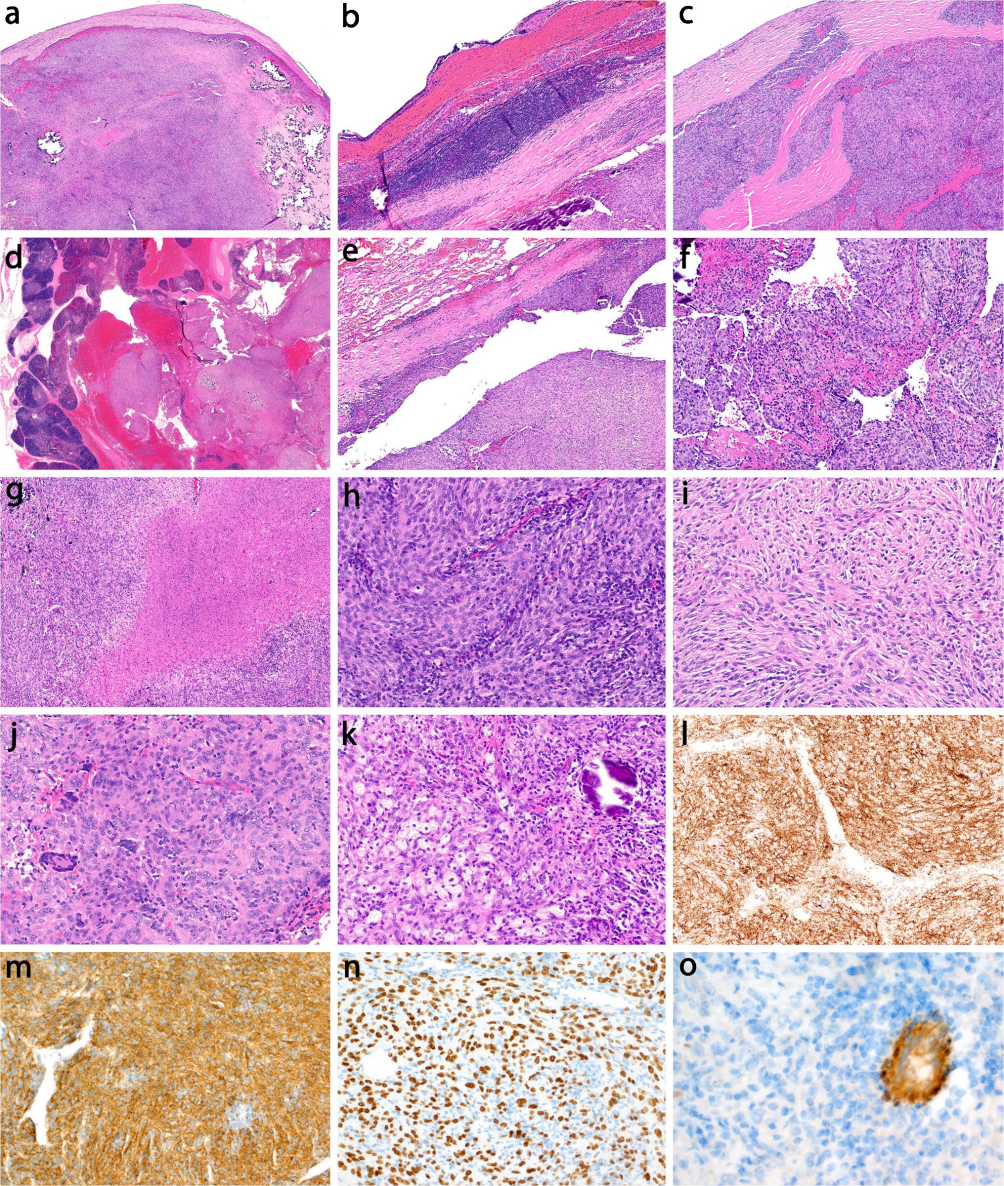
Light microscopic and immunohistochemical findings. (**a**) Well-circumscribed tumor with a thick fibrous capsule and extensive calcifications (H&E, × 4**). **(**b**) Pericapsular lymphoid cell aggregates (H&E, × 10). (**c**) Tongue-like tumor growth within the thick capsule (H&E, × 10). (**d**) Tumor intimately related to thymic tissue on left (H&E, × 2.5). (**e**) Tumor in relation to lung tissue with subcapsular clefting (H&E, × 10). (**f**) Dilated and cleft-like vascular spaces causing tumor fragmentation (H&E, × 20). (**g**) Foci of necrosis (H&E, × 20). (**h**) Bland ovoid to spindle cells in short fascicles with a prominent lymphoplasmacytic inflammatory infiltrate. Thin-walled vessels also present (H&E, × 40). (**i**) Spindle cell foci devoid of inflammation (H&E, × 40). (**j**) Scattered multinucleated giant cells (H&E, × 40). (**k**) Aggregates of foamy macrophages and a psammomatous calcification (H&E, × 40). (**l**) Diffuse HMB45 staining (× 40). (**m**) Diffuse cathepsin K staining (× 40). (**n**) Diffuse homogenous nuclear labeling for TFE3 protein (× 40). (**o**) Loss of nuclear staining for the C-terminus of YAP1 with retained expression in normal background stromal and vascular cells (× 40)

Histologically, the tumor was well circumscribed and largely enclosed by a thick fibrous capsule, accompanied by extensive stromal dystrophic calcifications with psammomatous forms (Fig. 2a). Pericapsular lymphoid cuffing and tongue-like protrusions of tumor within the capsule were identified (Fig. 2b,c). Thymic tissue was identified microscopically, showing direct tumor–thymus juxtaposition and focal loss of the fibrous capsule at the interface; the tumor also abutted lung parenchyma without evidence of infiltration (Fig. 2d,e). On low magnification, the tumor showed a solid to lobulated growth pattern with dilated hemangiopericytoma-like and cleft-like vascular spaces, conferring a propensity for fragmentation (Fig. 2e,f). Foci of necrosis were noted (Fig. 2g). The neoplasm consisted of a relatively monotonous population of medium-sized plump ovoid to spindle cells with fine chromatin, inconspicuous nucleoli, and moderately eosinophilic cytoplasm, arranged in sheets, short fascicles, and vague storiform areas, associated with delicate thin-walled vessels. Mitotic activity was inconspicuous (1 per 10 high-power fields). A prominent patchy lymphoplasmacytic inflammatory infiltrate was present throughout, raising consideration of inflammatory myofibroblastic tumor (IMT) (Fig. 2h,i). Scattered eosinophils, multinucleated giant cells and aggregates of foamy macrophages were also present (Fig. 2j,k). No melanin-type pigment was noted and there was no evidence of lipogenesis or tortuous dysplastic vessels.

## Immunohistochemistry

Immunohistochemically, the tumor cells showed diffuse cytoplasmic expression of the melanocytic markers HMB45 and pan-melanoma cocktail, in addition to diffuse expression of cathepsin K and TFE3 (Fig. 2l,m,n). Variable patchy expression of MiTF and h-caldesmon and very focal Melan-A/MART1 positivity was noted. Complete loss of C-terminus YAP1 staining was limited to the neoplastic cells with retained reactivity in the stromal background cells (Fig. 2o). All other markers were negative (S100, SOX10, smooth muscle actin, desmin, calponin, pan-CK, CK8/18, CK19, CK5/6, EMA, p63, p40, TdT, CD5, CD117, CD20, CD3, CD163, CD1a, chromogranin, CD56, OCT3/4, SALL4, CD34, ERG, myogenin, ALK, and TTF1).

## Molecular findings

RNA-based targeted next-generation sequencing (NGS) was performed on formalin-fixed paraffin-embedded tumor tissue utilizing the Archer FusionPlex Sarcoma Panel v2 (Integrated DNA Technologies, USA). Testing identified an in-frame *YAP1* exon 4 (NM_001282101.1) to *TFE3* exon 7 (NM_006521.4) fusion transcript (breakpoints: chr11:102,070,735 and chrX:48,891,384; coordinates based on the GRCh37/hg19 reference genome). Additionally, analysis using a second DNA-based cancer gene panel (Oncomine Comprehensive Assay v3, Thermo Fisher Scientific, USA) demonstrated no pathogenic variants in *TSC1/TSC2* and no additional clinically relevant alterations across 161 cancer-associated genes.

The patient remained disease-free at 4 months, with long-term surveillance recommended given the presence of concerning histological features.

## Discussion

In this report, we describe a rare case of *YAP1::TFE3*–rearranged PEComa occurring at an unusually young age (5 years), a presentation that has not been previously reported in this disease. *TFE3*-rearranged PEComas, first recognized around 2010 [2], represent a distinct molecular subset accounting for a small minority of PEComas; however, the identity and spectrum of *TFE3* fusion partners were incompletely defined in early studies. With the advent of next-generation sequencing, *SFPQ (PSF)* has emerged as the most frequent fusion partner, with reported cases spanning a broad anatomic distribution, including visceral and somatic soft-tissue sites [10, 11]. Additional *TFE3* fusion partners identified in PEComas include *NONO*, *ASPSCR1*, *PRCC*, and *DVL2*, among other rare partners described in isolated reports [4, 12–14]. Until recently, however, *YAP1* has not been reported as fusion partner in PEComa. This fusion was originally described in 2013 in a rare subset of epithelioid hemangioendothelioma characterized by distinctive histological and clinical features [15]. In 2021, Agaimy et al. reported recurrent *YAP1::TFE3* fusions in CCST-L that definitionally lacked myomelanocytic features [7]. Additionally, an unusual unclassified cutaneous fibromyxoid mesenchymal neoplasm carrying this fusion was reported [16]. The final landmark of this fusion was the recent study by Kojima et al. who described two cases of PEComas characterized by prominent associated inflammation [5]. Since then, *YAP1::TFE3*–rearranged “inflammatory spindle cell” PEComa has emerged as a distinct entity, with a total of eight molecularly confirmed cases reported to date, including the current case. Of these, five arose in the lung and three in non-visceral somatic soft tissues (Table 1). Collectively, the tumors appear to represent a clinicopathologic subset of PEComa with a predilection for the thoracic region and a characteristic histology composed predominantly of spindle cells arranged in short fascicles or vague storiform patterns, accompanied by prominent intratumoral chronic inflammatory infiltrates; two reported tumors, however, were predominantly epithelioid [17, 18]. The present case additionally demonstrated a thick fibrous capsule with focal lymphoid cuffing, features also reported by Malik et al. [19]. Notably, our tumor showed extensive stromal dystrophic calcifications which were radiologically apparent and have been previously documented in one case from the same study [19]. Across reported cases, other recurring features include well-circumscribed borders (7/8), abundant thin-walled vessels +/− vascular dilation (7/8), focal necrosis (4/8), foamy histiocytes (6/8), histiocytic giant cells (3/8), scattered eosinophils (4/8), focal cytoplasmic clearing (2/8), extensive cytoplasmic clearing (1/8), cytoplasmic melanin (2/8), and prominent nuclear atypia (1/8). In the current case, focal accentuation of vascular dilation contributed to tumor lobulation and fragmentation. Importantly, all reported tumors demonstrated melanocytic differentiation by immunohistochemistry, often accompanied by reduced expression of smooth muscle markers as described in other *TFE3*-rearranged PEComas [11, 13].

**Table 1:** Reports of molecularly confirmed *YAP1::TFE3*–rearranged PEComas in the English literature

Ref*	Age/sex	Site/size	Melanocytic IHC	Smooth muscle IHC	TFE3 IHC	Fusion	Treatment	Outcome/follow-up (months)
Kojima et al., 2025 [5]	61/M	Lung/ 3.1 cm	HMB45 + MelanA focal MiTF –	SMA – Desmin – h-caldesmon +	+	YAP1 ex7 – TFE3 ex7	Lobectomy	NED (32)
	68/M	Lung/ 2.8 cm	HMB45 focal MelanA focal	SMA focal Desmin – h-caldesmon focal	+	YAP1 and TFE3 breakapart by FISH	Lobectomy	NED (7)
Hsu et al., 2025 [17]	64/F	Lung/ 5.5 cm	HMB45 + MelanA – MiTF – Cathepsin K +	SMA focal Desmin –	+	YAP1 ex4 – TFE3 ex7	Wedge & lingula resection+ LN dissection	Satellite nodules at diagnosis, NED (3)
MacDonald et al., 2025 [18]	53/F	Lung/ 3.2 cm	HMB45 +MelanA − MiTF +	SMA focal Desmin – h-caldesmon –	+	YAP1 ex4 – TFE3 ex7	Lobectomy	NA
Malik et al., 2026 [19]	57/F	Vulva/ 4 cm	HMB45 + MelanA focal Cathepsin K focal	SMA + Desmin focal	NA	YAP1 ex4 – TFE3 ex7	NA	NED (1)
	51/F	Presacral/ 5 cm	HMB45 +	SMA + Desmin –	NA	YAP1 ex3 – TFE3 ex7	NA	NED (4)
	76/F	Thigh/ 3.8 cm	HMB45 + MelanA –	SMA + Desmin –	NA	YAP1 ex4 – TFE3 ex7	NA	NED (6)
Current case	5/F	Anterior mediastinum/ 8 cm	HMB45 + MelanA focal MiTF focal Cathepsin K +	SMA – Desmin – Calponin – h-caldesmon focal	+	YAP1 ex4 – TFE3 ex7	Excision with thymectomy	NED (4)

*M* male,* F *female,* IHC* immunohistochemistry,* NED *no evidence of disease,* NA *not available

*TFE3-rearranged PEComas with unknown gene partners have been excluded

As *YAP1::TFE3* is not entirely specific, interpretation of this gene fusion requires integration with histologic and immunohistochemical findings. CCST-L, first described by Falconieri et al. in 2013 [6], represents the most relevant differential diagnosis in the present case, given the thoracic location, morphologic overlap, and shared *YAP1::TFE3* fusion [7]. CCST-L is composed of epithelioid to spindle cells with clear to pale eosinophilic cytoplasm arranged in solid sheets or nests, with prominent vascularity, and is distinguished primarily by the absence of myomelanocytic or other lineage-specific differentiation. The precise relationship between *YAP1::TFE3*-rearranged PEComa and CCST-L remains unresolved and warrants further study. Inflammatory myofibroblastic tumor (IMT) was also considered, particularly given the patient’s age, pulmonary proximity, and prominent inflammatory infiltrate. However, the tumor lacked SMA expression, showed no ALK immunoreactivity, and did not harbor *ALK* rearrangements on molecular testing. Angiomatoid fibrous histiocytoma (AFH) was another diagnostic consideration, characterized by a multinodular growth of spindled to ovoid cells with pseudoangiomatous spaces and peripheral lymphoplasmacytic cuffing. Nevertheless, AFH typically expresses desmin, lacks melanocytic marker expression, and is characterized by *EWSR1* rearrangements involving members of the CREB gene family (*CREB1*, *ATF1*, or *CREM*), none of which were detected in this case.

Most reported *YAP1::TFE3*–rearranged inflammatory spindle cell PEComas were clinically indolent, with patients being disease-free at short-term follow-up, although at least one case has been classified as malignant [17]. According to the predictive criteria by Folpe et al. for PEComa (size > 5 cm, infiltrative growth, high nuclear grade, mitotic rate > 1/50 HPF, necrosis, and vascular invasion) [20], the current tumor demonstrates two worrisome features—large size and necrosis—suggestive of potential aggressive behavior. Notably, a recent study has shown that malignant *TFE3*-rearranged PEComas often harbor complex copy-number alterations [21], which were not identified in this case. The patient remains free of disease at 4-month postoperative follow-up. Delineation of the full biological potential of these rare tumors requires long-term follow-up studies.

In summary, we report a pediatric case of intrathoracic *YAP1::TFE3*–rearranged inflammatory spindle cell PEComa showing clinicopathologic features consistent with those described in the original report by Kojima et al. and subsequent reports. Notably, the tumor was unusually large and exhibited extensive calcifications, further expanding the morphologic spectrum of this rare PEComa variant. Loss of the YAP1 C-terminus protein in this case suggests the potential value of combined YAP1 and TFE3 IHC to screen for the fusion in potential candidates.

## Data Availability

The datasets generated during and/or analyzed during the current study are not publicly available but are available from the corresponding author on reasonable request.
